# Nursing Homes as Insurers? The Effect of Provider-Led Institutional Special Needs Plans

**DOI:** 10.1093/geroni/igaf122.1015

**Published:** 2025-12-31

**Authors:** Amanda Chen, David Grabowski

**Affiliations:** University of Southern California, Los Angeles, California, United States; Harvard University, Cambridge, Massachusetts, United States

## Abstract

Institutional Special Needs Plans (I-SNPs) are a type of Medicare Advantage (MA) plan that allow insurers in MA to differentiate their benefits exclusively for long-term residents of nursing homes. When I-SNPs were first offered in 2006, the early I-SNP models were insurer-led as the plan was primarily at-risk for long-stay residents’ Medicare spending. Today however, nursing homes have increasingly been entering the I-SNP market with I-SNP models differentiated based on the level of risk borne by the insurer and nursing home. We use a difference-in-differences design to estimate the effect of offering a provider-led I-SNP on enrollment and quality of care. Using Medicare claims and the Minimum Data Set resident assessments from 2004-2021, we find that offering a provider-led I-SNP led to a 17.0 percentage point increase in I-SNP enrollment among facility residents within four years relative to control nursing homes. We also estimate that offering provider-led I-SNPs significantly decreased hospitalizations and increased the use of hypnotic drugs and reporting of pressure ulcers by nursing homes. Relative to the baseline mean among treated nursing homes, we found relative declines of 18.2% for hospitalizations and increases of 30.3% for hypnotic drug use and 15.6% for pressure ulcers. These results suggest that risk bearing by nursing homes in the form of provider-led I-SNPs may successfully reduce utilization, but with unclear implications for quality and competition.

